# Supplementary material for: Effect of Buddleja cordata Leaf Extract on Diabetic Nephropathy in Rats
Source: Int J Mol Sci. 2024 Oct 24;25(21):11432. doi: 10.3390/ijms252111432 (PMC11546301; doi:10.3390/ijms252111432)
Supplement: Supplementary file 1 [file ijms-25-11432-s001.zip › ijms-3136877-supplementary.pdf]

Table S1. Comparison glycemia, body weight, water and food ingestion, and urinary volume: control (c), untreated diabetes mellitus (DM), diabetes mellitus treated with vitamin E (Vit E 250 mg/kg), diabetes mellitus treated with captopril (25 mg/kg) and diabetes mellitus treated with methanol extract of B. cordata (50, and 100 mg/kg), (EMB). *p <0.05 Control vs treatment, and # p<0.05 DM vs treatment.

		DM +				
Parameter	control	DM	Vit E	CAP	EMB 50	EMB 100
Glycemia (mg/dL)	100 ± 3	500 ± 14*	482 ± 12*#	493 ± 20	498 ± 25	450 ± 23#
Body weight (g)	398 ± 7	320 ± 16	345 ± 14	355 ± 8	256 ± 3#	345 ± 10 #
Water ingestión (mL/24h)	34 ± 4	144 ± 9*	103 ± 2*#	98 ± 10*	110 ± 23	153 ± 7
Urinary volume (mL/24h)	23 ± 3	102 ± 4*	66 ± 8#	66 ± 3#	78 ± 5#	124 ± 3
